# Knowledge and Preventive Practices Regarding Diabetes Mellitus Among Non-Diabetic Adults in Kamrup Rural District of Assam: A Cross-Sectional Study

**DOI:** 10.7759/cureus.99498

**Published:** 2025-12-17

**Authors:** Darshana Hazarika, Imran Khan, Mangala Lahkar

**Affiliations:** 1 Nursing, Sharda School of Nursing Science and Research, Sharda University, Greater Noida, IND; 2 Nursing, North East Medical Care (NEMCARE) Institute of Nursing Sciences, Guwahati, IND; 3 Pharmacology, Srimanta Sankaradeva University of Health Sciences, Guwahati, IND

**Keywords:** adults, diabetes mellitus, knowledge, practice, prevention

## Abstract

Background: Diabetes mellitus (DM) is a chronic condition that is distinguished by abnormally elevated blood glucose levels.

Aim and objective: The aim of this present study is to determine the knowledge and preventive practices regarding diabetes mellitus among non-diabetic adults in Kamrup Rural (R) District of Assam

Methodology: Descriptive, cross-sectional design was adopted in the present study to evaluate knowledge and preventive practices regarding diabetes mellitus among non-diabetic adults. The data was collected using a structured questionnaire. Spearman’s correlation, Fisher’s exact test and Chi-square tests were employed for analyzing the data.

Results: Of the adults surveyed, 52% demonstrated adequate knowledge of diabetes mellitus, 47% had moderately adequate knowledge, and only 1% had inadequate knowledge.. The preventive practices were found to be good in 55% of adults, but 45% showed poor practices. There was a moderate, statistically significant correlation between knowledge and preventive practices regarding diabetes mellitus (Spearman’s ρ=0.496, p< 0.001). A significant relation was found between knowledge and age, but no demographic factor was found to relate to practices.

Conclusion: The study finally concludes that the evident knowledge-practice gap underscores the necessity of health interventions that go beyond mere education to effectively motivate and allow behavioural change, particularly targeting different demographic groups.

## Introduction

The prevalence of non-communicable diseases (NCDs) has continued to be a global issue over the years. Regardless of their developmental stage, diabetes is one of the NCDs that are progressively presenting challenges to countries [1,2]‌. Diabetes mellitus (DM) is a chronic condition characterised by significantly elevated blood glucose levels. Type 2 diabetes mellitus (T2DM) and type 1 diabetes mellitus (T1DM) are the most common variants of diabetes. Hyperglycemia, insulin resistance, and relative insulin secretion deficit are the hallmarks of T2DM [3,4].

Since T2DM is the most widespread type of the disease, 90-95% of people with diabetes have it. Its association with morbidity and mortality, which has an impact on the person’s general health and well-being, makes it a serious public health concern. This disease is more common in younger age groups, affecting both adults and adolescents [5]. Due to the fast changes in way of living brought about by urbanisation, the risk factors for diabetes, hypertension and obesity have grown [6]. Approximately 537 million adults in the age group of 20-79 years around the world had diabetes in 2021 (global prevalence, 10.5%), which is anticipated to rise to 783 million (12.2%) by 2045 [7]. Among these, 45% were undiagnosed, with the greatest prevalence in low and middle-income countries [8]. Sedentary lifestyles, cigarette smoking, alcohol consumption, stressful situations, and a high-fat diet are also recognised as contributing factors to the development of Diabetes Mellitus.

Several individuals with DM are more susceptible to variety of short, as well as long term complications, which frequently result in permanent deaths [9]. Prolonged hyperglycaemia can lead to severe complications and organ dysfunction, specifically in the kidney, blood vessels, and eyes. Peripheral neuropathy, nephropathy, retinopathy, and autonomic neuropathy are significant long-term complications of diabetes [10]. According to a study done by Misra et al., a rapid surge in obesity across developing countries correlates with the rise in diabetes and cardiovascular issue-related deaths [11].

Assessment of an individual’s understanding of their illness has a beneficial effect on health education interventions. As a result, it is imperative to evaluate the knowledge and practices of persons with diabetes mellitus. Sufficient knowledge does not necessarily result in effective self-management practices or insufficient knowledge that leads to inadequate self-care practices [12,13]‌. Knowledge is the most formidable weapon in the fight against DM. Research proved that knowledge is a contributing factor for one to have a good attitude and proper practise to prevent DM [14].

A study carried out by Herath et al. revealed that most of the participants (77%) had moderate knowledge, while (39%) had above moderate knowledge regarding diabetes mellitus. The level of education demonstrated a significant and positive association with knowledge (p=0.001); however, the associations of gender and age with knowledge were statistically not significant. In terms of practices majority (80%) did not engage in regular exercise activities [15].

Research has shown that individuals who are knowledgeable regarding diabetes self-care achieve greater prolonged glycemic control. This awareness of glycemic control enables individuals to recognise risk factors that have been linked to diabetes and encourages them to pursue proper medical care, thus enabling the effective management of the disease. Emphasising knowledge and preventive strategies among non-diabetic adults is fundamental to public health initiatives aimed at combating diabetes. By providing adults with the appropriate information and resources before disease onset, we can avert significant human suffering, mitigate substantial economic burdens, and promote longer, healthier lives. Therefore, the study intended to evaluate the knowledge and preventive practice regarding diabetes mellitus among the adult who are not diabetic.

## Materials and methods

Research design

A descriptive, cross-sectional design was adopted in the present study to evaluate knowledge and preventive practices regarding diabetes mellitus among non-diabetic adults

Ethical consideration

Ethical permission was obtained from the Institutional Ethics Committee (NGI/Ethics/PhD/2025/002) of the NEMCARE group of institutions, Mirza, Kamrup. The subjects were informed about the purpose and method used in the present study. Oral and written consent was taken from the subjects.

Study setting, sample size and sampling technique

Data were collected between July and August 2025. This study involved 220 non-diabetic adults who resided in the Sarpara and Kochpara villages of Kamrup, Rural district, Assam. The total number of eligible adults available in the selected two villages (Sarpara and Kochpara) during the data collection period was identified through local community records. Out of this population, 220 participants were selected based on their willingness to participate, accessibility, and suitability according to the inclusion-exclusion criteria.

Inclusion criteria

This study recruited participants who were non diabetic, willing to participate and who were in the age group between 30 and 60 years

Exclusion criteria

Unwilling participants, adults with diabetes mellitus, pregnant women and individuals with a history of mental illness, psychiatric issues or any serious health issues were excluded from this study.

Data collection methods and instruments used

Researchers developed a structured questionnaire organised in English and later translated into the local language (Assamese) to collect data from the participants. The questionnaire was divided into three sections. Section I consist of a total of seven questions regarding demographic characteristics of the samples; Section II includes 12 questions to evaluate the knowledge about diabetes mellitus; and Section III includes six questions to evaluate the practice on prevention of diabetes mellitus among non-diabetic adults. Each correct answer in the questionnaire carries 1 mark, and an incorrect answer is marked as 0 points. For the knowledge questions, scoring was categorised as 1-4 as inadequate knowledge, 5-8 as moderately adequate knowledge and 9-12 as adequate knowledge. Regarding practice questions, scoring was categorised as 0-3 as poor practice and 4-6 as good practice (Appendix A, B, C).

Validity and reliability

The questionnaire was distributed among seven experts for establishing content validity. Reliability of the baseline demographic proforma was calculated by intra ratter method (r=1). Reliability of the knowledge and practice questionnaire was determined using the split-half technique, and the 'r' value was determined using the Spearman formula (r=0.7 and 0.8, respectively).

Statistical analysis

The statistical analyses were performed using SPSS software (IBM Corp., 2011. IBM SPSS Statistics for Windows, Version 20.0). Descriptive statistics, including frequencies and percentages, were used to analyse demographic characteristics and levels of knowledge and preventive practices regarding diabetes mellitus. Spearman’s rank correlation coefficient was applied to assess the relationship between knowledge and preventive practices among non-diabetic adults. Fisher’s exact test and the Chi-square test were used to determine the association between knowledge and preventive practices and selected demographic characteristics.

## Results

Table 1 presents that the majority of the 220 individuals in the study were middle-aged or older. Males made up a larger percentage, and about half of them were married, but a sizable portion were single. While most participants had only completed elementary or high school, a tiny percentage had higher education, indicating a variety of educational backgrounds among the participants. The majority of respondents were non-vegetarians, and the majority had a family history of diabetes. The percentage who had ever participated in any diabetes management course or program was quite small.

**Table 1:** Frequency and percentage distribution of demographic characteristics.

Demographic characteristics	Frequency (%)
Age	
30-40 years	82 (37)
41-50 years	98 (45)
51-60 years	40 (18)
Gender	
Male	131 (60)
Female	89 (40)
Marital status	
Married	108 (49)
Unmarried	98 (44)
Widow	10 (5)
Divorced	4 (2)
Educational qualification	
No formal education	6 (3)
Primary school	79 (36)
Middle school	16 (7)
High school	104 (47)
Higher secondary	9 (4)
Graduation and above	6 (3)
Family history of DM	
Yes	167 (76)
No	53 (24)
Food habit	
Vegetarian	60 (27)
Nonvegetarian	160 (73)
Attended training/programme on management of DM	
Yes	58 (26)
No	162 (74)

Table 2 indicates that most participants established good awareness of the basic aspects of diabetes. A large majority correctly identified diabetes as a condition of inadequate insulin production and recognised that excessive sugar intake can contribute to its development. Awareness of genetic risk, however, was comparatively lower, with only about half acknowledging this factor.

**Table 2:** Participants response (frequency and percentage) for knowledge regarding of diabetes mellitus.

Items	Frequency (%)	
	Yes	No
Q1 DM is a condition of insufficient insulin production	194 (88)	26 (12)
Q2 Eating too much sugar can cause DM	184 (84)	36 (16)
Q3 DM is not curable	125 (57)	95 (43)
Q4 DM is hereditary	115 (52)	105 (48)
Q5 Sedentary lifestyle can lead to diabetes	158 (72)	62 (28)
Q6. Frequent urination is the symptoms of DM	184 (84)	36 (16)
Q7 Diabetic is manageable by diet & exercise	196 (89)	24 (11)
Q8 DM can be treated by medication	114 (52)	106 (48)
Q9 DM can be treated by insulin injection	154 (70)	66 (30)
Q10 DM effect many body parts	198 (91)	22 (10)
Q11 DM increase the risk of other disease	121 (55)	99 (45)
Q12 DM reduces life expectancy	157 (71)	63 (27)

**Figure 1:**
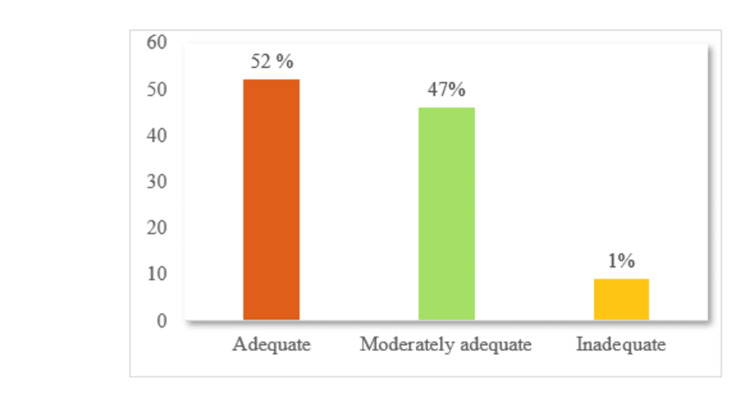
Percentage distribution of knowledge regarding diabetes mellitus.

Figure 1 shows that just over half of the adults demonstrated adequate knowledge of diabetes mellitus, while nearly an equal proportion had moderately adequate knowledge. Only a very small fraction of participants exhibited inadequate knowledge, indicating generally favorable awareness levels in the study population.

Table 3 indicates that most of the participants engaged themselves in several recommended preventive practices for diabetes. A large percentage stated that they avoid fatty foods, try to maintain a healthy weight and regularly consume fibre-rich foods, vegetables and fruits. More than half reported checking their blood sugar at least annually, although fewer participants engaged in daily physical exercise.

**Table 3:** Participants response (frequency and percentage) for practice regarding prevention of diabetes mellitus.

Items	Frequency (%)	
	Yes	No
1. Do you try to avoid fatty foods?	194 (88)	26 (12)
2. Do you try to maintain a healthy body weight?	184 (84)	36 (16)
3. Do you perform 30-60-minute physical exercise daily?	124 (57)	95 (43)
4. Do you smoke?	115 (52)	105 (47)
5. Do you check your blood sugar regularly (at least annually)?	158 (72)	62 (28)
6.. Do you take food containing fibres, vegetables and fruits everyday	196 (89)	24 (11)

**Figure 2:**
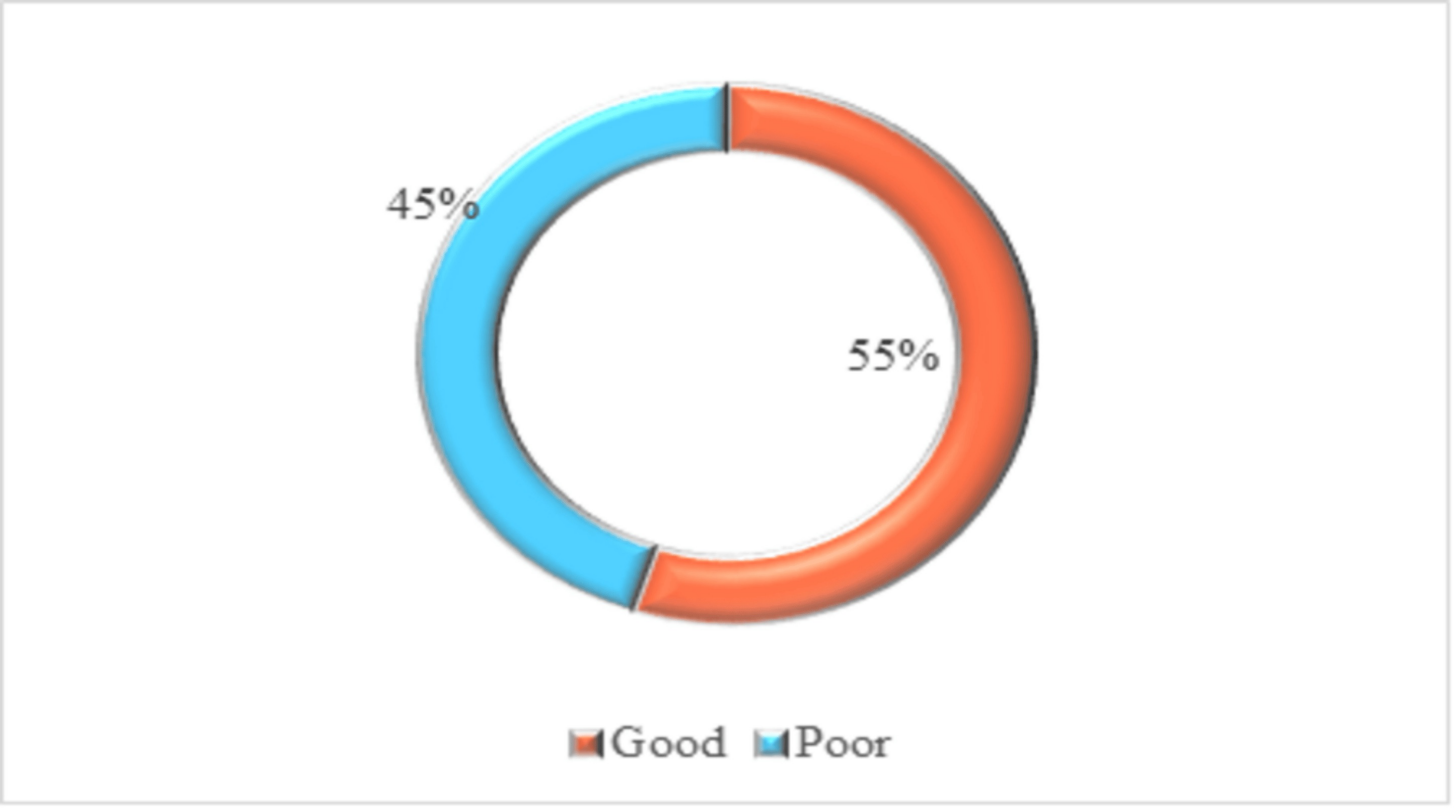
Percentage distribution of practice regarding prevention of diabetes mellitus.

Figure 2 shows that slightly more than half of the adults demonstrated good preventive practices toward diabetes mellitus, while the remaining participants exhibited poor practice levels, indicating scope for improvement in overall health-promoting behaviours.

Table 4 demonstrates a moderate, statistically significant correlation between knowledge and preventive practice regarding diabetes mellitus (Spearman’s ρ=0.496, p< 0.001). This suggests that individuals with higher knowledge about diabetes tend to engage in better preventive practices.

**Table 4:** Correlation between knowledge and preventive practice regarding diabetes mellitus among non-diabetic adults. Significant at p< 0.001.

		Knowledge score	Practice score
Knowledge score	Spearman’s rho	1	0.496
	Sig (2-tailed)	-	0.000
Practice score	Spearman’s rho	0.496	1
	Sig (2-tailed)	0.000	-

Table 5 shows the association between participants’ knowledge regarding diabetes mellitus and their demographic characteristics. The analysis exposed a significant association between age and knowledge level (p=0.021), indicating that knowledge varied meaningfully across different age groups. Participants in the younger and middle-aged categories appeared more likely to have adequate knowledge compared to older adults.

**Table 5:** Association between knowledge regarding diabetes mellitus with their selected demographic characteristics. *Indicates significant at p< 0.05

Demographic characteristics	Adequate	Moderately adequate	Inadequate	Fisher’s exact	p-value
Age				9.75	0.021*
30-40 years	53	29	0		
41-50 years	43	54	1		
51-60 years	19	20	1		
Gender				4.14	0.972
Male	69	61	1		
Female	46	42	1		
Marital status				4.49	0.822
Married	58	49	1		
Unmarried	48	49	1		
Widow	7	3	0		
Divorced	2	2	0		
Educational qualification				8.66	0.731
No formal education	49	54	1		
Primary school	45	33	1		
Middle school	8	8	0		
High school	3	3	0		
Higher secondary	7	2	0		
Graduation and above	3	3	0		
Family history of DM				3.58	0.511
Yes	89	77	1		
No	26	26	1		
Food habit				0.428	0.937
Vegetarian	31	29	0		
Non vegetarian	84	74	2		
Attended training/programme on management of diabetes mellitus				8.87	0.066
Yes	29	39	1		
No	86	64	1		

No significant associations were found for gender, marital status, educational qualification, family history of diabetes, food habits, or attendance in diabetes-related training programmes (p> 0.05). These findings suggest that, except for age, the other demographic factors did not have a measurable influence on participants’ knowledge levels regarding diabetes mellitus.

Table 6 presents the association between participants’ preventive practices for diabetes mellitus and their demographic characteristics. The results show that none of the demographic variables, including age, gender, marital status, educational qualification, family history of diabetes, food habit or attendance in diabetes-related training, were significantly associated with preventive practice levels (p> 0.05).

**Table 6:** Association between preventive practice regarding diabetes mellitus with their selected demographic characteristics. Significant at p< 0.05.

Demographic characteristics	Good	Poor	χ²	p-value
Age			4.56	0.102
30-40 years	38	44		
41-50 years	61	37		
51-60 years	22	18		
Gender			0.826	0.662
Male	72	59		
Female	49	40		
Marital status			3.23	0.357
Married	60	48		
Unmarried	55	43		
Widow	3	7		
Divorced	3	1		
Educational qualification			5.95	0.311
No formal education	61	43		
Primary school	45	34		
Middle school	8	8		
High school	2	4		
Higher secondary	2	7		
Graduation and above	3	3		
Family history of DM			0.192	0.909
Yes	91	76		
No	30	23		
Food habit			0.370	0.543
Vegetarian	31	29		
Non vegetarian	90	70		
Attended training/programme on management of diabetes mellitus			0.825	0.662
Yes	38	31		
No	83	68		

These findings indicate that preventive behaviors related to diabetes were comparable across all demographic groups, suggesting that factors such as age, education, and lifestyle habits did not significantly influence engagement in good or poor preventive practices.

## Discussion

These findings of the current investigation indicate that 115 (52%) of the adults had adequate, 103 (47%) had moderately adequate, and 2 (1%) had inadequate knowledge regarding diabetes mellitus. This finding is comparable with the study reported by Baig M et al wherein 66.86% of non-diabetic adults had a good knowledge and 33.14% had poor knowledge regarding diabetes mellitus [16]. Results of the investigation are also comparable with the research carried out by Dinesh et al., in which only 24% of the participants had good knowledge regarding diabetes [17].

The present study showed that out of 220 participants (88%), respondents actively try to avoid fatty foods. 184 (84%) reported that they try to maintain a healthy weight. 124 (57%) of respondents perform 30-60 minutes of physical exercise daily. Notably, 115 (52%) reported that they do not smoke. Most participants (158, 72%) do not check their blood sugar regularly (at least annually). The majority (196, 89%) of participants consumed fibre-rich foods, vegetables and fruits daily. These findings were consistent with the research study done by Baig M et al, in which 459 individuals (38%) consumed oily foods less frequently, while only 338 individuals (28%) and 153 individuals (12.7%) engaged in physical activities for 30-60 minutes per day on a frequent or very frequent basis, respectively. The majority of participants, 890 (73.7%), were tobacco smokers and received their blood pressure checks frequently (704, 58.3%).

Additionally, these findings are comparable to those reported by Alsous et al., in which only 62.3% of participants engaged in regular physical exercise. More than half of the participants had not undergone blood glucose testing in the preceding year, and approximately 45.3% reported consuming refined sugar [18].

In the present study, among the 220 non-diabetic adults, 121 (55%) demonstrated good preventive practices, while 99 (45%) exhibited poor practices regarding diabetes mellitus prevention. These findings contrast with those reported by Wolde W. et al., in which 64.6% of participants had poor practices, and only 35.4% had good practices related to diabetes prevention [19]. The current study demonstrates a moderate positive and statistically significant correlation between knowledge and preventive practice regarding diabetes mellitus (Spearman’s ρ=0.496, p< 0.001).

The result of this study is comparable with the findings reported by Vrinda et al., where a weak but significant positive correlation was found between knowledge and practice (r=0.349) [20]. The current study shows the association between participants’ knowledge regarding diabetes mellitus and their demographic characteristics. The analysis exposed a significant association between age and knowledge level (p=0.021). This study’s outcome contrasts with the findings reported by Herath et al., who observed that diabetes knowledge was not significantly associated with age or gender but showed a significant association with educational level (p=0.001) [15].

Limitations

This study’s limitations is its reliance on a purposive sampling method to select 220 participants from only two villages in the Kamrup (R) district of Assam. This substantially restricts the applicability of the findings to the general populace of adults.

## Conclusions

The presentation showed that a majority of adults exhibited sufficient knowledge about diabetes mellitus, but a substantial proportion of adults failed to practice preventive strategies effectively. Despite differences in preventive knowledge among different age groups, no variation in preventive practices was found among different demographic groups. A discrepancy between knowledge and practice stipulates the need for strategies that enhance not only awareness but also sustainable lifestyle changes. Overall, the findings emphasise the importance of community-focused interventions that strengthen both understanding and preventive actions to reduce the future burden of diabetes.

## Appendices

Appendix A

**Table 7:** Demographic proforma used in the study.

Variables	Response options
Age (years)	30–40 ☐ 41–50 ☐ 51–60 ☐
Gender	Male ☐ Female ☐ Transgender ☐
Marital status	Single ☐ Married ☐ Widowed ☐ Divorced ☐
Educational qualification	No formal education ☐Primary school ☐Middle school ☐High school ☐Higher secondary ☐Graduation and above ☐
Dietary pattern	Vegetarian ☐ Non-vegetarian ☐
Family history of diabetes	Yes ☐ No ☐
Attended training/program on diabetes management	Yes ☐ No ☐

Appendix B

**Table 8:** Structured knowledge questionnaire on diabetes mellitus.

Item No.	Knowledge statement	Yes	No
1	Diabetes mellitus is a condition of insufficient insulin production	☐	☐
2	Eating excessive sugar can cause diabetes mellitus	☐	☐
3	Diabetes mellitus is not curable	☐	☐
4	Diabetes mellitus is hereditary	☐	☐
5	Frequent urination is a symptom of diabetes mellitus	☐	☐
6	A sedentary lifestyle can lead to diabetes	☐	☐
7	Diabetes can be managed by diet and exercise	☐	☐
8	Diabetes can be treated with medication	☐	☐
9	Diabetes can be treated with insulin injections	☐	☐
10	Diabetes affects multiple body systems	☐	☐
11	Diabetes increases the risk of other diseases	☐	☐
12	Diabetes reduces life expectancy	☐	☐

Appendix C

**Table 9:** Structured practice questionnaire on diabetes prevention.

Item No.	Practice statement	Yes	No
1	Do you try to avoid fatty foods?	☐	☐
2	Do you attempt to maintain a healthy body weight?	☐	☐
3	Do you perform 30–60 minutes of physical exercise daily?	☐	☐
4	Do you smoke?	☐	☐
5	Do you check your blood sugar regularly (at least once a year)?	☐	☐
6	Do you consume fibre-rich foods, vegetables, and fruits daily?	☐	☐

## References

[1] Animaw W, Seyoum Y (2017). Increasing prevalence of diabetes mellitus in a developing country and its related factors. PLoS One.

[2] Al-Wagdi BE, Al-Hanawi MK (2024). Knowledge, attitude and practice toward diabetes among the public in the Kingdom of Saudi Arabia: a cross-sectional study. Front Public Health.

[3] Alaofè H, Hounkpatin WA, Djrolo F, Ehiri J, Rosales C (2021). Knowledge, attitude, practice and associated factors among patients with type 2 diabetes in Cotonou, Southern Benin. BMC Public Health.

[4] Almousa AY, Hakami OA, Qutob RA (2023). Knowledge, attitude, and practice toward diabetes mellitus and their association with socioeconomic status among patients with type 2 diabetes mellitus in Saudi Arabia. Cureus.

[5] Al-Aboudi IS, Hassali MA, Shafie AA (2016). Knowledge, attitudes, and quality of life of type 2 diabetes patients in Riyadh, Saudi Arabia. J Pharm Bioallied Sci.

[6] Hazarika D, Khan I, Lahkar M (2025). Knowledge on importance of yoga therapy among type II diabetic adults of Mirza, Kamrup (R), Assam: a descriptive study. Imran Khan.

[7] Sun H, Saeedi P, Karuranga S (2022). IDF Diabetes Atlas: Global, regional and country-level diabetes prevalence estimates for 2021 and projections for 2045. Diabetes Res Clin Pract.

[8] Ogurtsova K, Guariguata L, Barengo NC (2022). IDF diabetes Atlas: Global estimates of undiagnosed diabetes in adults for 2021. Diabetes Res Clin Pract.

[9] Olokoba AB, Obateru OA, Olokoba LB (2012). Type 2 diabetes mellitus: a review of current trends. Oman Med J.

[10] BeliyaLuxmi D, Singh SH, Singh SJ (2019). Prevalence of type 2 diabetes in northeast India: a review. Int J Curr Res.

[11] Misra A, Khurana L (2008). Obesity and the metabolic syndrome in developing countries. J Clin Endocrinol Metab.

[12] ElSayed NA, Aleppo G, Aroda VR (2023). Facilitating positive health behaviors and well-being to improve health outcomes: standards of care in diabetes-2023. Diabetes Care.

[13] Maduemezia U, Variava E, Moloantoa T (2024). Knowledge, attitude, and practices related to diabetes among patients with type 2 diabetes mellitus at Tshepong Hospital. J Endocrin Met Diab South Africa.

[14] Mahadeva R, Zin T, Win-RN KK, Subramaniam ALS, Shan BT, Mogan APK, Ismail BAS (2018). Cross-sectional study on knowledge, attitude and practice regarding diabetes mellitus among medical and non-medical students. Res J Pharm Technol.

[15] Herath HM, Weerasinghe NP, Dias H, Weerarathna TP (2017). Knowledge, attitude and practice related to diabetes mellitus among the general public in Galle district in Southern Sri Lanka: a pilot study. BMC Public Health.

[16] Baig M, Alzahrani S, Abualhamael S, Alotaibi A, Alharbi M, Almohammadi T, Alkaabi T (2023). Diabetes mellitus knowledge, attitudes, preventive practices and associated factors among a sample of adult Non-Diabetic Saudi Residents. Diabetes Metab Syndr Obes.

[17] Dinesh PV, Kulkarni AG, Gangadhar NK (2016). Knowledge and self-care practices regarding diabetes among patients with Type 2 diabetes in Rural Sullia, Karnataka: A community-based, cross-sectional study. J Family Med Prim Care.

[18] Alsous M, Abdel Jalil M, Odeh M, Al Kurdi R, Alnan M (2019). Public knowledge, attitudes and practices toward diabetes mellitus: A cross-sectional study from Jordan. PLoS One.

[19] Wolde W, Demeke AD, Atle D, Girma D, Hailu S (2025). Knowledge, attitude, and practice towards diabetes mellitus among Chiro town population, Eastern Ethiopia. BMC Public Health.

[20] Vrinda Vrinda, Bhat S, Anjana KS, Kavya N, Monica KP (2024). The correlation between knowledge, attitude, and practice related to diabetes involving ayurveda and yoga in two ayurveda and allopathy hospitals of Bengaluru. J Int Health Sci.

